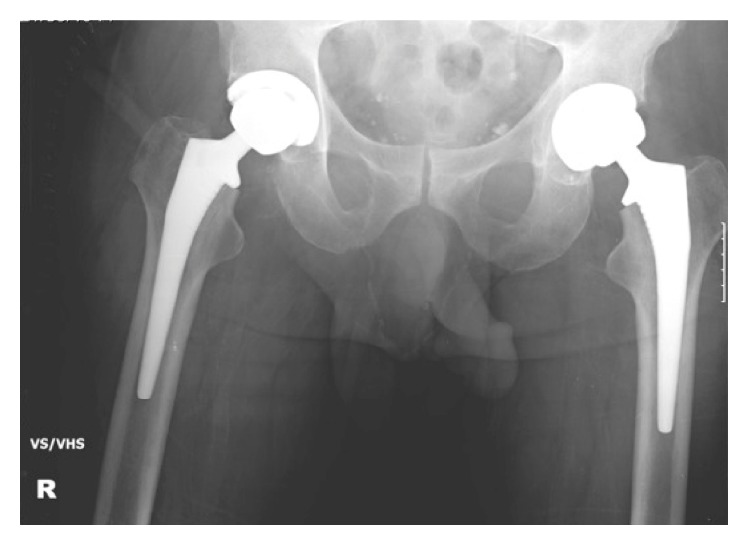# Bilateral Neck of Femur Fractures in a Bilateral Below-Knee Amputee: A Unique Case

**DOI:** 10.1155/2016/7083671

**Published:** 2016-01-06

**Authors:** Hannah R. Lancer, Peter Smitham, Pinak Ray

**Affiliations:** Plastic Surgery Department, Royal Free Hospital, Pond Street, London NW3 2QG, UK

## Abstract

According to the National Hip Fracture Database, over 64,000 patients were admitted with a hip fracture across England, Wales, and Northern Ireland in 2013, but very few are bilateral, and there are no current cases in the literature of bilateral neck of femur fractures in a patient with bilateral below-knee amputations. We present a case of a 69-year-old bilateral below-knee amputee male admitted to the emergency department with bilateral hip pain and radiological evidence of bilateral displaced neck of femur fractures. The patient subsequently underwent synchronous bilateral total hip replacements under general anaesthetic and an epidural and then went on to make a full recovery. He was discharged 27 days after arrival in hospital. Outpatient follow-up at 3 months has shown that the patient has returned to a similar level of preinjury function and is still able to carry out his daily activities with walking aids and bilateral leg prostheses.

## 1. Introduction

Hip fractures are particularly common, with 64,838 cases being admitted to hospital in 2013 [1]. The age-range with the highest incidence for neck of femur fractures is 80–89 years (47.2%), and for ages 60–69, the incidence was 8.8% in 2013 [1], yet simultaneous bilateral neck of femur fractures are rare and usually occur in high-energy trauma, underlying bone disease or seizure disorders [2].

We highlight an unusual case presentation of bilateral displaced femoral neck fractures in a below-knee amputee, and his successful outcome. This case report can serve as a reference to other surgeons should a similar case arise in their own practice, and we review the current literature on bilateral hip fractures and hip fractures in amputees.

## 2. Case Presentation

A 69-year-old gentleman presented to the emergency department with bilateral hip pain. He described two falls in the past week, although he was still able to bear weight and continue with his daily activities.

The patient had previously undergone bilateral below-knee amputations; the right lower leg was amputated 6 years prior to this presentation as a result of Charcot foot complicated by diabetes mellitus, with subsequent osteomyelitis, whilst the left lower leg was amputated 1 year before this presentation, following severe water scalding and subsequent failed skin grafting and gangrene.

The patient's other comorbidities included an ileostomy for ulcerative colitis (panproctocolectomy in 1969), chronic kidney disease (baseline creatinine of 200 umol/L), type 2 insulin-dependent diabetes mellitus, ischaemic heart disease, and Coeliac disease.

His medication on admission included omeprazole, bisoprolol, sodium bicarbonate, aspirin, iron sulphate, adcal D3, trimethoprim, novorapid, and glargine.

Although a bilateral amputee, the patient was still extremely active and continued to work as an optometrist at his local hospital with the aid of bilateral leg prostheses.

## 3. Investigations

**Figure 1:**
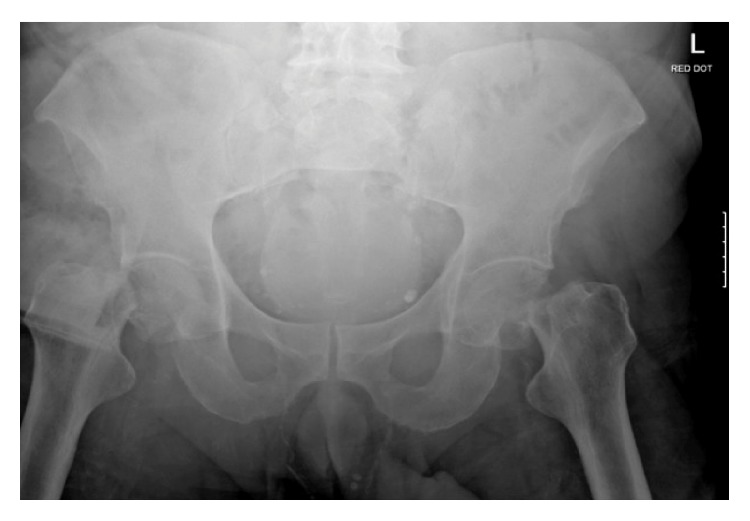


In the emergency department, X-rays of the pelvis revealed bilateral displaced intracapsular neck of femur fractures (Figure 1).

## 4. Treatment

On the day of admission, following informed consent and under a general anaesthetic and an epidural, the patient underwent bilateral uncemented total hip replacements via a posterior approach. A metal-on-polyethylene implant was used for both hips; a 36 mm sized head was adhered to the femoral stem, with a 52 mm acetabular shell (Figure 2). The stability of both sides was confirmed intraoperatively. Closure was performed in layers with clips to the skin, and no intraoperative complications were encountered. Of note at operation, an old healed right sided greater trochanteric fracture was noted. Theatre time totaled 4 hours and 23 minutes.

Postoperatively, the patient was transferred to the Intensive Care Unit for 2 days and during his stay developed a metabolic acidosis with multiple episodes of hyperkalaemia. This was treated medically, and the patient's kidney function returned to baseline.

Rehabilitation is important in any patient undergoing such a procedure, but particularly so in a patient with prior mobility concerns.

Specific challenges in physiotherapy included retraining and reeducating the patient as hip precautions had to be introduced postoperatively. These included not crossing the legs or flexing the hip joint to more than 90 degrees. The patient used to transfer himself from chair to bed and would fit his own prostheses; these activities had to be addressed thoroughly postoperatively by the physiotherapy team in order to try and prevent hip dislocation. Solutions included help from his wife in attaching his prostheses, and changing the patient's usual transfer practice.

At discharge, the patient was referred back to the Royal National Orthopaedic Hospital in the outpatient setting for new prostheses. It was there where his prostheses had been fitted initially and physiotherapy had subsequently taken place. This was of particular importance, as it was thought that the reason for the patient's falls had occurred due to poorly fitting prostheses (the patient described using 3 socks over his amputations in order for the prostheses to fit correctly).

## 5. Outcome

The patient continued to be reviewed in the outpatient setting following discharge at day 27, and he still mobilises with the aid of two sticks. The patient states that his mobility has not been impaired, and he is still continuing to work as an optometrist.

## 6. Discussion

A PubMed search using the terms “bilateral hip fracture OR bilateral femoral neck fracture OR bilateral neck of femur fracture” AND “bilateral amputation/amputee” identified only 6 papers, of which none related exactly to this case.

Low-energy neck of femur fractures are common, but no cases in the literature have been described for bilateral neck of femur fractures in a patient with bilateral below-knee amputations, and therefore there is no set protocol for such a patient.

The majority of bilateral hip fractures are as a result of high-energy impact, with one study reporting the incidence of bilateral hip fracture presenting to a level 1 trauma center as 8 patients over a 10-year period [3]. In our case we describe a male with two clear episodes of trauma to the hip within a week, and although bilateral neck of femur fractures are rare, it has been described [2].

However, there are reports in the literature of bilateral femoral neck fractures occurring as a result of predisposing factors, such as convulsions secondary to hypocalcaemia [4], osteomalacia [5], osteoporosis, and renal osteodystrophy [6, 7]. In addition to trauma, bilateral fractures due to epilepsy and electrocution have been described [8].

Data from the National Hip Fracture Database Anaesthesia Sprint Audit of Practice (ASAP) state that patients should be offered the choice of spinal or general anaesthetic after discussing risks and benefits with the patient. Nationally, in 2013, about 50% of patients underwent a general anaesthetic, 44% underwent a spinal anaesthetic, and 3% endured both a general and spinal anaesthetic. The remainder had “other” anaesthesia, mainly as local anaesthesia. There are no set protocols regarding anaesthesia, and preference varied markedly between hospitals [9].

National guidelines for femoral neck fractures state that patients should have surgery performed either the day of or the day after admission [1, 10] and correctable comorbidities should be identified and amended promptly to avoid delays in surgery, which can lead to increased morbidity and mortality. Intraoperative positioning of the patient with a neck of femur fracture in a bilateral amputee has been described, but only when a traction table has been required, which is not necessary for a total hip replacement [11].

Management of this particular patient adhered to these guidelines, and it was decided by the team that the use of two uncemented total hip replacements would be implemented. Benefits of uncemented implants include less need for revision due to aseptic loosening, but drawbacks are a higher incidence of periprosthetic fracture [12]. There is no difference in infection or dislocation [13]. Conversely, a recent meta-analysis for femoral neck fractures in the elderly population by Li et al. found that cemented hemiarthroplasty, compared against uncemented hemiarthroplasty, can provide better hip function, lower residual pain, and less implant related complications [14].

McBryde et al. described elective one-stage bilateral metal-on-metal total hip replacements compared to two-stage for bilateral hip disease, and results did not show any difference in complication rate or transfusion requirements [15]. In addition, the one-stage group had a reduced hospital stay, and hospital costs were reduced without significantly altering the patients' recovery.

Of the few articles on this subject, one paper described 5 patients who had one-sided total hip-arthroplasty for displaced subcapital neck of femur fractures with below-knee amputations and all of these patients made a good recovery with return to prefracture level of activity [16].

For this case specifically, the patient had some significant comorbidities, and there tends to be an increased risk of morbidity following femoral neck fracture in elderly men with renal dysfunction, both in the acute and in chronic phase, which is related to the eGFR and creatinine levels: length of hospital stay is negatively associated with the eGFR [17]. The patient in this case was admitted with chronic kidney disease, with a baseline creatinine level of 200 umol/L and eGFR of 31 mls/min/1.73 m^2^ (stage 3b chronic kidney disease). His inpatient stay totaled 27 days, where the national average length of stay is 20 days [1].

In addition, the risk of hip fracture for men with type 2 diabetes mellitus is increased compared to those without the disease [18]. We know that this patient did suffer two episodes of trauma as a cause for his bilateral fractures, but this comorbidity may well have had an association with the proven fractures.

In conclusion, bilateral total hip replacements can be undertaken in a patient with bilateral below-knee amputations successfully, and, with good rehabilitation, the patient can return to preoperative mobility.

## Figures and Tables

**Figure 2:**